# Fecal microbiota from chronically stressed donors transfers behavioral and prefrontal cortex gene expression alterations to healthy recipient mice

**DOI:** 10.1016/j.ynstr.2026.100843

**Published:** 2026-08-04

**Authors:** Lucas Hassib, Raphael de Andrade Morraye, Wilson Araújo Silva, Francisco Silveira Guimarães, Frederico Rogério Ferreira

**Affiliations:** a Department of Pharmacology, Ribeirão Preto Medical School, University of Sao Paulo, Ribeirão Preto, SP, 14049-900, Brazil; b Oswaldo Cruz Foundation, Institute Oswaldo Cruz, Rio de Janeiro, RJ, Brazil; c Department of Genetics, Ribeirão Preto Medical School, University of Sao Paulo, Ribeirão Preto, SP, 14049-900, Brazil

**Keywords:** Microbiota, Stress, Fecal microbiota transplantation, Gut-brain axis

## Abstract

Over the past decades, alterations in gut microbiota composition have emerged as a key contributor to the neurobiology of stress-related psychiatric conditions, including Major Depressive Disorder (MDD). Clinical and preclinical evidence consistently demonstrates correlational associations indicating that the gut microbiota not only responds to stress but also modulates the host stress response, thereby influencing behavioral domains relevant to depressive symptomatology. However, relatively few studies have investigated the causal relationship between stress-induced microbiota alterations and stress-associated behavioral impairments. Here, we examined the role of the enteric microbiota in stress-related behaviors using fecal microbiota transplantation (FMT) from animals exposed to a Chronic Unpredictable Stress (CUS) protocol to reproduce stress-associated behavioral phenotypes in recipient mice. Donor animals subjected to a 28-day CUS protocol exhibited behavioral alterations across despair-like, anhedonia-like, and anxiety-like domains, as assessed by the Novelty Suppressed Feeding, Sucrose Spray, Tail Suspension, and Elevated Plus Maze tests. FMT from stressed donors successfully transferred similar behavioral alterations to recipient animals. Dysregulated expression of synapsin 1 (Syn1) and oligodendrocyte transcription factor 1 (Olig1) was observed in the prefrontal cortex of both donor and recipient animals. In the hippocampus, CUS-exposed donors showed reduced Olig1 and a trend toward reduced Sox2 expression, whereas no significant hippocampal changes were detected in FMT recipients, suggesting that prefrontal, but not hippocampal, molecular alterations are preferentially transferable via the gut microbiota. Sequencing of the V4 region of the 16S rRNA gene revealed increased relative abundance of *Lachnospiraceae* and *Parabacteroides* and reduced *Lactobacillales* in both stressed donors and FMT recipients. In contrast, recipients of control microbiota exhibited higher levels of genera commonly associated with gut homeostasis, including *Bacteroides*, *Ligilactobacillus* and *Lactobacillus*. Together, our findings demonstrate that stress-induced microbiota alterations can mediate the transmission of stress-associated behavioral and molecular alterations, particularly in the prefrontal cortex, supporting the relevance of the gut–brain axis in stress-related disorders, while the underlying signaling mechanisms remain to be elucidated.

## Highlights

• Chronic unpredictable stress changes gut microbiota profile.
• Alterations in gut microbiota composition persist in FMT recipients and resemble donor microbial signatures.
• Fecal microbiota transplantation from stressed donors reproduces behavioral impairments in non-stressed recipients.
• Stress-related dysbiosis transfers alterations in prefrontal cortex gene expression, including reduced Syn1 and Olig1.

## 1 Introduction

Major depressive disorder (MDD) is a profoundly debilitating stress-related psychiatric condition characterized by persistent low mood, anhedonia, cognitive impairment, and vegetative symptoms, including sleep and appetite disturbances (Malhi and Mann, 2018). Anxiety frequently co-occurs with MDD, compounding its clinical burden (Zhou et al., 2017). Globally, MDD remains one of the leading causes of disability (Yang et al., 2024), with a complex etiology that reflects the interplay of genetic predispositions and environmental factors (Otte et al., 2016), particularly stress, which is widely recognized as a key contributor to both depression and gut dysbiosis (i.e., a harmful imbalance or alteration of gut microorganisms that can result in disease) (Aroniadis and Grinspan, 2024). Furthermore, recent clinical and preclinical research advances have highlighted the intricate relationship between gut microbiota and MDD (Nikolova et al., 2021). A bidirectional communication mediates this interplay between the central nervous system (CNS) and the gut, collectively referred to as the gut-brain axis (Cryan et al., 2019; Pedrazzi et al., 2024; Hassib et al., 2024), which operates through multiple pathways, including immune signaling, metabolites and hormones release into the systemic circulation, and direct neural connections such as the vagus nerve (Hassib et al., 2023, 2024; Pedrazzi et al., 2023).

Clinically, a compelling link between gut health and CNS functioning in depression was first observed when patients diagnosed with MDD were found to have a higher prevalence of gastrointestinal symptoms such as chronic diarrhea and constipation compared to non-depressed individuals (Sharma et al., 2022; Ballou et al., 2019). Moreover, these patients often exhibit an increased incidence of gastrointestinal disorders, including irritable bowel syndrome (Ruan et al., 2023). On the preclinical side, pivotal research in 2004 already demonstrated that germ-free animals, lacking any gut microbiota, exhibit heightened sensitivity to stress compared to conventionally colonized animals (Sudo et al., 2004). Since this discovery, numerous microorganisms have been identified as modulators of stress responses, being able to influence several behaviors in animals (Xu et al., 2020, 2024). For example, certain strains like *Lactobacillus* have been found to protect the host against stress (Xu et al., 2020), whereas others, such as *Parabacteroides*, appear to potentiate stress responses (Xu et al., 2024). Supporting this connection, patients with MDD typically exhibit increases in groups such as *Parabacteroides* genera and *Lachnospiraceae* family and decreases in beneficial genera like *Lactobacillus* and *Prevotella*, suggesting a gut microbial signature associated with stress response (McGuinness et al., 2022; Aizawa et al., 2016).

While these correlational studies have elucidated a consistent association between gut microbiota alterations and stress in patients and animal models, empirical investigations into how shifts in microbiota composition might causally impair CNS function and ultimately drive behavioral deficits remain sparse. Therefore, this study aims to address this gap by examining whether stress-associated microbiota alterations induced via Fecal Microbiota Transplantation (FMT) from donors subjected to the Chronic Unpredictable Stress (CUS) protocol can reproduce stress-associated behavioral deficits in recipient animals. Specifically, we investigate whether this stress-induced microbiota shift can lead to stress-related behaviors and whether these impairments reflect abnormalities in the Central Nervous System, assessed through gene markers associated with synaptogenesis, oligodendrogenesis, and neuroplasticity.

## 2 Materials and methods

### 2.1 Experimental animals and housing

The Institute of Science and Technology in Biomodels (ICTB- Fiocruz) provided Swiss mice, which were 6 weeks old upon arrival. Only male animals were used in the present study. Following a two-week acclimatization period, these mice were assigned to their respective experimental groups at the age of 8 weeks. Animals were typically housed in groups of four per home cage to simulate a standard living environment. However, mice designated for the CUS protocol were housed individually to prevent the mitigating effects of sociability on stress responses, as documented in previous studies (Ma et al., 2011). Detailed information regarding the number of animals utilized in each experimental analysis is provided in the respective figures' captions.

### 2.2 Chronic Unpredictable Stress protocol

The CUS protocol involves a series of moderate, random stressors applied daily over four weeks, as detailed in Supplementary Table 1. This protocol was adapted from the methodologies from Katz and Hersh (1981) and Monteiro et al. (2015), comprising the following stressors:

*Forced swimming*: animals were placed individually in containers with approximately 1.5 L of water for 5 min;
*Tilted cage*: animals were kept in their cages, inclined at a 45° angle for 1 h without bedding to prevent stable plane formation;
*Hot air stream*: each animal was exposed to a stream of hot air (45-50 °C) from a hairdryer for 5 min, aimed at the back while avoiding direct eye exposure;
*Bedding removal*: animals spent the night in cages without any bedding;
*Wet bedding*: animals were left overnight with excessively wet bedding, to the point of dripping when squeezed;
*Food deprivation*: animals underwent 14 h of food deprivation overnight in their cages;
*Cycle alternation* - animals were kept in dark conditions during the daytime and in brightly lit conditions at night, disrupting their circadian rhythms;
*Restraint* - animals were confined in a 50 ml plastic tube with breathing openings on both sides for 1 h.

Mice were subjected to the CUS regimen daily throughout the fecal collection period for FMT, except on days designated for behavioral testing. Behavioral assessments were performed immediately after the fourth week of CUS exposure, as illustrated in Fig. 1. In contrast, animals in the control (CTL) group were not exposed to stress and were instead handled gently daily.

**Fig. 1:**
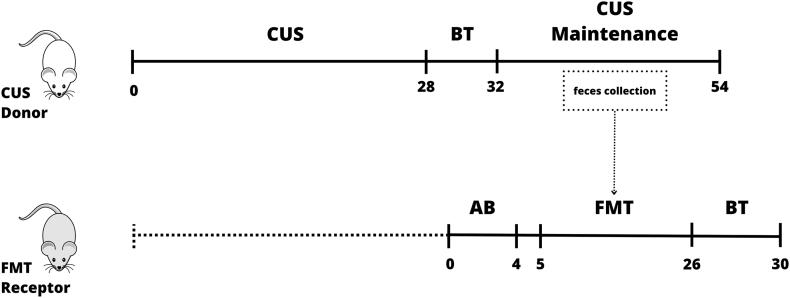
CUS and FMT protocols. Fecal Microbiota from CUS and control mice were collected and used in the FMT protocol daily for 21 days. BT = Behavioral Tests. AB = Antibiotic.

### 2.3 Antibiotic treatment

To reduce interference from the existing gut microbiota and promote colonization by the introduced microorganisms, mice designated for FMT were treated with the injectable antibiotic Baytril (enrofloxacin, Bayer Inc.), adapted from Johnson et al. (2004). The antibiotic was administered subcutaneously at a dosage of 5 mg/kg body weight daily for four consecutive days, after which the FMT protocol was initiated.

FMT was initiated 24 h after the last antibiotic administration. Based on pharmacokinetic data in mice reporting an elimination half-life of enrofloxacin of approximately 89 min (Bregante et al., 1999), this interval corresponds to approximately 16 half-lives, ensuring negligible plasma drug concentrations at the time of transplantation. Importantly, the antibiotic pre-treatment was applied uniformly to all recipient animals, regardless of the donor group, ensuring that any residual microbiota-level effects of antibiotic exposure were equivalent across groups and did not constitute a source of differential bias.

### 2.4 Fecal microbiota transplantation

Fecal microbiota inocula were prepared by pooling 3 g of freshly collected feces from all donor animals within each group (CUS or CTL) on the morning of each transplantation day. Pooling was chosen to standardize the microbial inoculum across recipients and to minimize donor-to-donor variability, consistent with previously published FMT protocols (Kelly et al., 2016; Zheng et al., 2016; Li et al., 2019). We acknowledge that this strategy precludes individual donor–recipient matching, a limitation discussed in the Limitations section.

Fecal suspensions were prepared under aerobic conditions in 20 ml of sterile phosphate-buffered saline and immediately administered to recipients by oral gavage to minimize oxygen exposure. We acknowledge that the absence of strictly anaerobic preparation conditions may have introduced a bias against the survival of oxygen-sensitive obligate anaerobes, and this is discussed as a limitation below. Nevertheless, 16S rRNA analysis of recipients revealed successful colonization by known obligate anaerobes (e.g., *Lachnospiraceae*, *Parabacteroides*), indicating that at least a portion of the anaerobic community was functionally transferred.

The mixture was then filtered through a sieve with a mesh size suitable to remove larger particulate matter, ensuring a uniform suspension. The resultant fecal solution was administered to the recipient mice via oral gavage using a stainless-steel Aton needle, bent at a 9-degree angle, and attached to a cannula with a distal diameter of 1.2 mm. Recipient animals were assigned to one of two parallel groups: the CUS-FMT group, which received microbiota from CUS donors, and the CTL-FMT group, which received microbiota from control donors and served as the control for the FMT procedure. Each recipient was inoculated with 5 μl of the solution per gram of body weight daily, continuing for 21 days, as illustrated in Fig. 1.

### 2.5 Behavioral tests

All behavioral tests were conducted during the light phase of the light-dark cycle, specifically in the morning hours. Before each test, animals were allowed to acclimate in the testing room for 1 h. The testing environment was sound-attenuated and temperature-controlled at 25 ± 1 °C, with a luminosity level maintained at 60 lx. The test equipment was thoroughly sanitized with 70% alcohol between sessions to ensure cleanliness. All sessions were recorded, and the tapes were subsequently analyzed by a trained observer blinded to the group assignments.

#### 2.5.1 Novelty Suppressed Feeding (NSF)

To assess anxiety and anhedonic-like behavior, mice were food-deprived for 24 h and then placed in an open rectangular acrylic box (28 × 17 × 13 cm) with a food pellet positioned centrally. Testing commenced with each mouse placed at one corner facing the wall. The primary behaviors recorded were the latency to approach the food and to commence eating (Bodnoff et al., 1988).

#### 2.5.2 Sucrose spray (SS)

As an additional measure of anhedonic-like behavior, the sucrose spray test involved placing mice in a clean cage devoid of bedding. A 10% sucrose solution was sprayed onto the head and dorsal fur, and grooming behavior was observed for 5 min. The frequency and the latency to start grooming within the interval were quantified (Ferreira et al., 2015).

#### 2.5.3 Elevated Plus Maze (EPM)

Anxiety-like behavior was also evaluated using the elevated plus-maze. This apparatus consists of two open arms (30 × 5 × 0.25 cm) and two closed arms (30 × 5 × 15 cm), with a central platform (5 × 5 cm) connecting them, set at a height of 45 cm from the floor. Mice were placed on the central platform facing a closed arm, and their activity during a 5-min session was recorded. Behaviors analyzed included the percentage of time spent in open arms, the number of risk assessments, and entries into closed arms (Pellow and File, 1986).

#### 2.5.4 Tail suspension (TS)

Each mouse was suspended by the tail using a 15 cm piece of tape to assess despair behavior and observed over 5 min. Immobility time, defined as the absence of paw movements or escape-oriented actions, was meticulously recorded (Steru et al., 1985).

### 2.6 Quantitative real-time PCR (qRT-PCR)

RNA was extracted from flash-frozen samples of the prefrontal cortex and hippocampus using the TRIzol reagent (Invitrogen), according to the manufacturer's protocol. The extracted RNA was then reverse-transcribed into cDNA using a High-Capacity cDNA Reverse Transcription Kit (Applied Biosystems). Quantitative real-time PCR (qRT-PCR) was performed to evaluate the relative mRNA expression levels of Syn1, Olig1, and Sox2 using specific primer pairs: Syn1 (F: *5′-CACCGACTGGGCAAAATACT-3*'; R: *5′-TCCGAAGAACTTCCATGTCC-3′*), Olig1 (F: *5′-GCCCCACCAAGTACCTGTCTC-3'*; R: *5′-GGGACCAGATGCGGGAAC-3′*), and Sox2 (F: *5′- AACGCCTTCATGGTATGGTC-3*; R: *5′-TCTCGGTCTCGGACAAAAGT-3′*) These assays were conducted on a StepOne Real-Time PCR System (Thermo Fisher Scientific) utilizing Power SYBR Green Master Mix (Thermo Fisher Scientific) according to the manufacturer's protocol (Ferreira et al., 2020). Total RNA normalization was based on the Ct values of GAPDH housekeeping gene (F: 5′-*TCCCTGAGCTGAACGGGAAG*-3'; R: 5′-*GGAGGAGTGGGTGTCGCTGT*-3′) (Chapman and Waldenström, 2015). Each sample was run in triplicate to ensure reproducibility, and relative expression changes were calculated using the 2−ΔΔCt method, as detailed in the referenced protocol (Ferreira et al., 2020).

### 2.7 16S ribosomal RNA (rRNA) gene amplicon sequencing

Fecal samples were collected from the cages of both donors (CUS and CTL) and recipient (CUS FMT and CTL FMT) mice one day before the behavioral testing, ensuring three cages per group. These samples were immediately preserved in 99% ethanol and stored at −80 °C. DNA extraction was performed using the QIAmp Power Fecal Pro DNA Kit (QIAGEN) following the manufacturer's instructions. Fecal samples for 16S rRNA sequencing were collected at the cage level (three cages per group, each containing four animals), rather than at the individual animal level. This approach was chosen due to the use of pooled fecal material from the donors' cages as the source for the FMT inoculum and to match the microbiota analyses with the pooling strategy used in the transplantation protocol.

Libraries were prepared to target the V4 region of the 16S ribosomal RNA gene using the 515F and 806R primer sequences (515F: *5′-TCGTCGGCAGCGTCAGATGTGTATAAGAGACAGGTGCCAGCMGCCGCGGTAA-3'*; 806R: *5′-GTCTCGTGGGCTCGGAGATGTGTATAAGAGACAGGGACTACHVGGGTWTCTAAT-3′*). These libraries were constructed using the Nextera XT DNA Library Preparation Kit (Illumina), which includes Illumina adaptors and molecular barcodes.

Sequencing was conducted on an Illumina MiSeq platform using a MiSeq Reagent Kit v2 (500 cycles) for 2 × 250 paired end reads (Sgritta et al., 2019). The resulting sequences, in FastQ format, were processed using the QIIME2 pipeline (version 2020.2)(Bolyen et al., 2019) for alignment and demultiplexing. Quality control was performed on the demultiplexed sequences using the RCPP package of the DADA2 software version 1.28.0 to filter out chimeric sequences (Callahan et al., 2016). Amplicon Sequence Variants (ASVs) from the V4 region were identified and matched to an optimized version of the SILVA Database (Quast et al., 2013). Data visualization and statistical analysis of the microbiome data were performed using the MicrobiomeAnalyst platform (Dhariwal et al., 2017). Data filtering and normalization adhered to standard platform criteria. Specifically, a low-count filter was applied, retaining features with a minimum count of 4 and a prevalence in at least 20% of samples. Additionally, a low-variance filter was used to exclude the bottom 10% of features based on interquartile range variability. Cumulative Sum Scaling (CSS) normalization was implemented to account for compositional differences in sequencing depth. Statistical analyses included the Kruskal-Wallis test for alpha diversity, permutational multivariate analysis of variance (PERMANOVA) for beta diversity, and differential abundance analysis using Linear Discriminant Analysis (LDA) Effect Size (LEfSe). The LEfSe analysis applied a logarithmic LDA score threshold of 2.0 and a significance level of P = 0.1.

### 2.8 Statistical analysis

The results are presented as the mean ± standard error of the mean (SEM). Data from behavioral tests and gene expression analyses involving microbiota donors and FMT recipients were analyzed using a two-tailed unpaired Student's t-test. This test was applied when the data met the assumptions of normality, verified by the Shapiro-Wilk test, and homogeneity of variance, which was assessed using Levene's test. Statistical analyses were performed with Prism software (version 8.0.2, GraphPad Software Inc., 2019). For datasets that did not meet the criteria for normal distribution or homogeneous variance, the two-tailed Mann-Whitney *U* test was used. Statistical significance was defined as p < 0.05.

## 3 Results

### 3.1 Behavioral, molecular, and microbiome effects of CUS protocol

In the TST, animals subjected to the CUS protocol exhibited prolonged immobility compared to the control group (p = 0.0078, Fig. 2A). Stressed mice also took longer to initiate facial grooming (p = 0.0152) and exhibited reduced grooming behavior (p = 0.0023) compared to controls (Fig. 2B and C). Although not reaching statistical significance, the NSF test showed a trend toward increased latency to begin eating among stressed animals compared to controls (Fig. 2D and E).

**Fig. 2:**
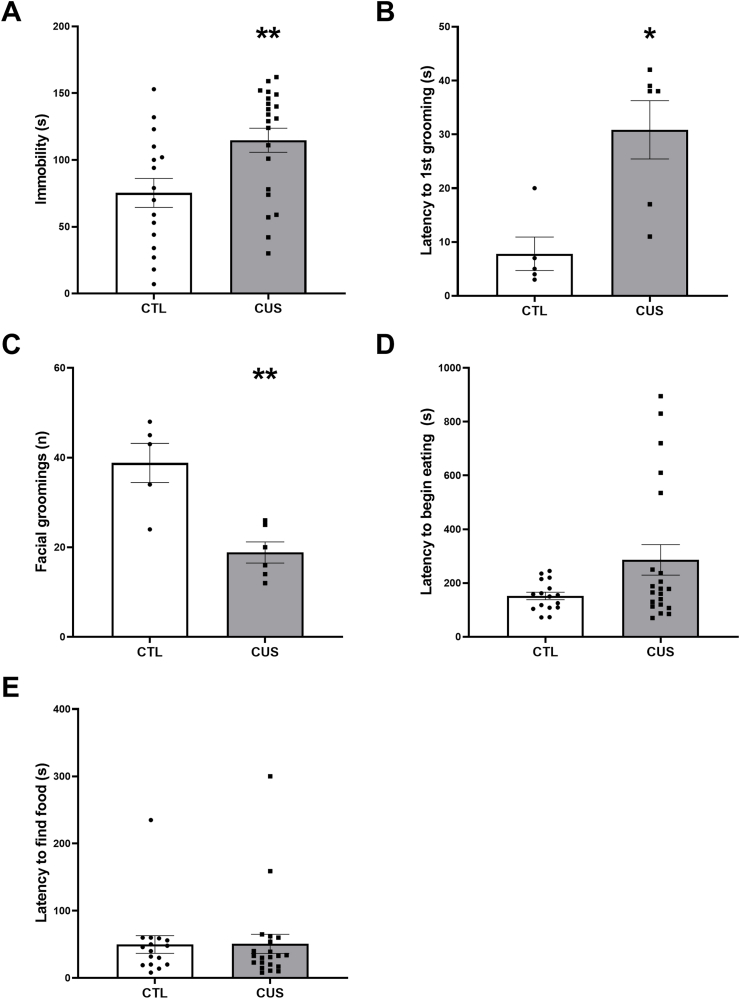
Effect of the CUS protocol on behaviors related to depressive-like behaviors in microbiota donors' mice. (A) Tail Suspension test; A = Immobility (s). (B-C) Sucrose Spray test; B = Latency to first grooming (s); C = number of facial groomings (n). (D-E) Novelty Suppressed Feeding test; D = Latency to begin eating (s); E = Latency to find food (s). Bars represent M±SEM; individual data points are shown. * Indicates P < 0.05, ** indicates P < 0.01. N Tail Suspension; Novelty Suppressed Feeding = 16-21; N Sucrose Spray = 5-6.

**Fig. 3:**
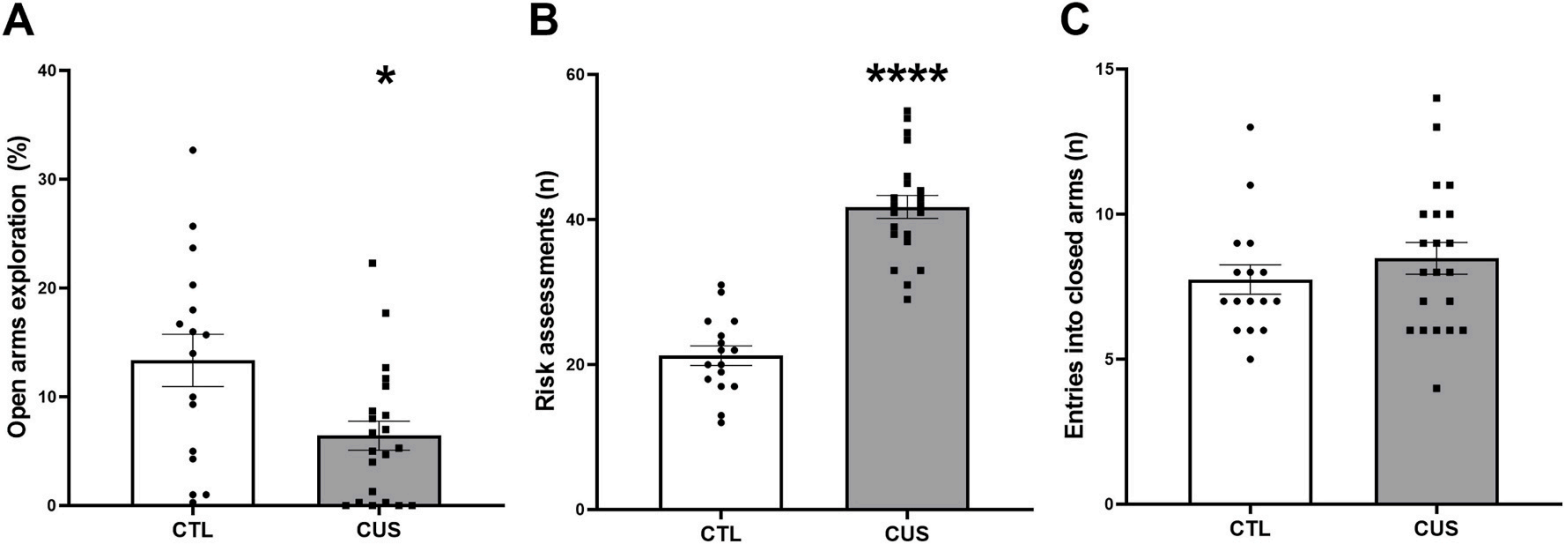
Effect of the CUS protocol on behaviors related to anxiety behaviors in microbiota donors mice. (A-C) Elevated Plus Maze test; A = Open arms exploration time (%); B = Risk assessment (n); C = Entries into closed arms (n). Bars represent M±SEM; individual data points are shown. * Indicates P < 0.05, **** indicates P < 0.0001. N = 16-21.

In the EPM test, the CUS protocol significantly decreased the exploration time in the open arms (Fig. 3A) and increased risk assessment behaviors (p < 0.0001; Fig. 3B) compared to the control animals. There was no effect on the number of entries into the closed arms (p = 0.3519; Fig. 3C).

**Fig. 4:**
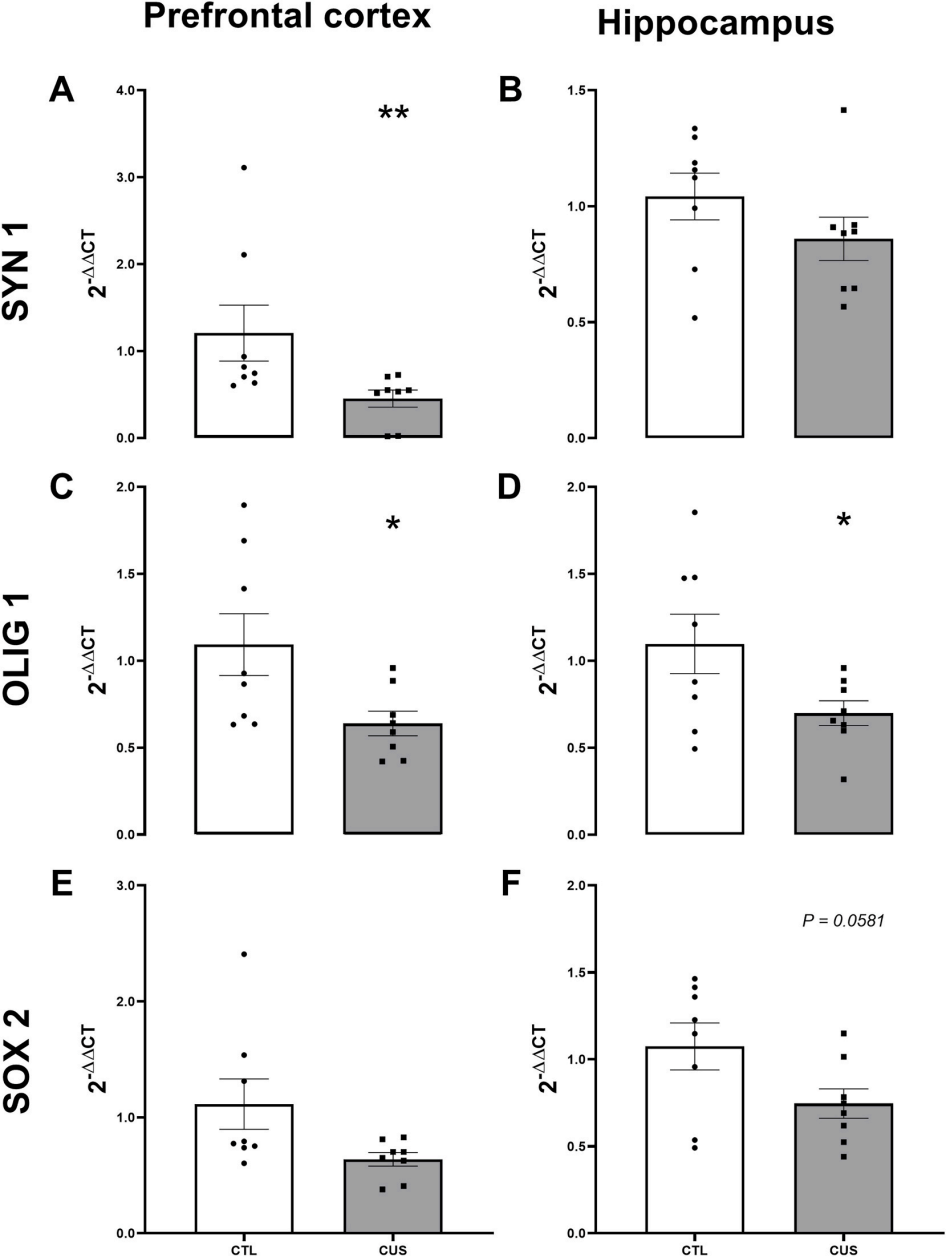
Effect of the CUS protocol on cortical and hippocampal gene expression in microbiota donors' mice. (A-B) Synapsin 1 (Syn1) expression (2-ΔΔCT). (C-D) Oligodendrocyte Transcription Factor 1 (Olig1) expression (2-ΔΔCT). (E-F) SRY-box 2 (Sox2) expression (2-ΔΔCT). Bars represent M±SEM; individual data points are shown. * indicates P < 0.05; ** indicates P < 0.01. N = 8,8.

The animals in the CUS group also exhibited a reduction in the expression of Syn1 (p = 0.0047) and Olig1 (p = 0.0325) mRNA in the prefrontal cortex compared with the non-stressed group. In the hippocampus, the CUS protocol reduced the expression of Olig1 (p = 0.0493) and Sox2 mRNA (p = 0.0581, Fig. 4).

**Fig. 5:**
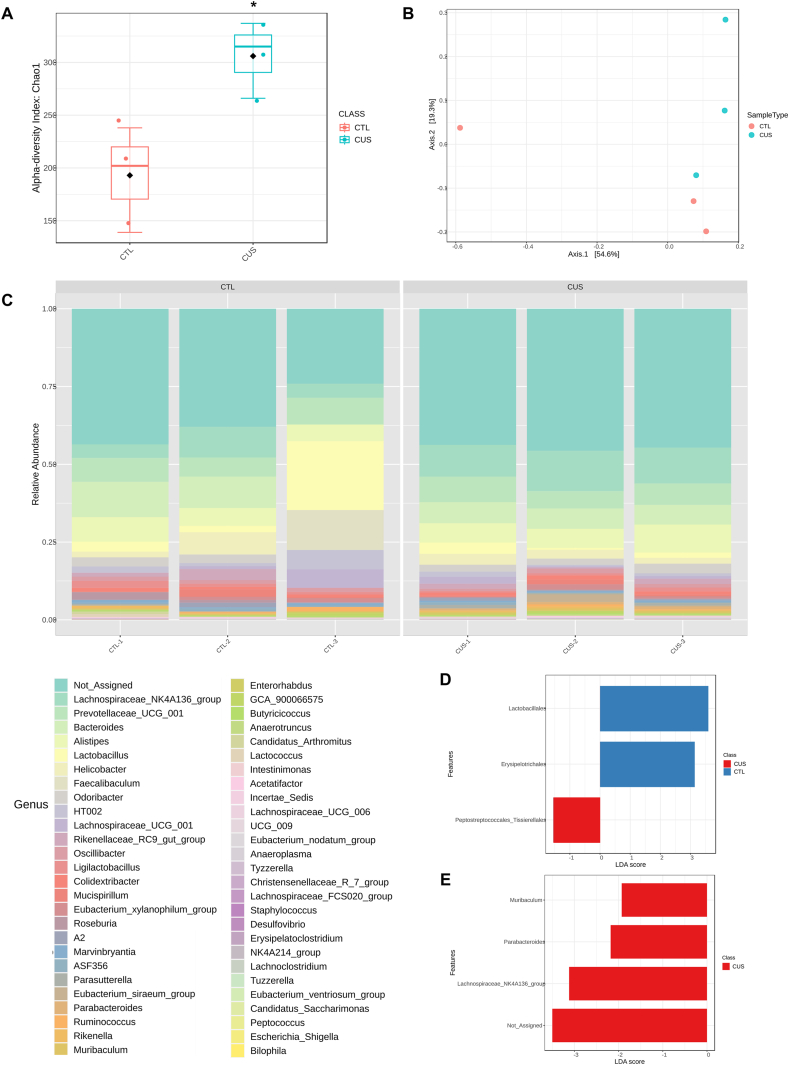
Effect of the CUS protocol on the fecal microbiota composition in microbiota donors' mice. (A) Alpha diversity according to the Chao1 index; individual data points are shown. (B) Beta diversity expressed in the Principal Coordinate Analysis (PCoA) plot with Bray-Curtis dissimilarity. (C) Stacked bar charts showing the actual relative abundance of bacterial genera in Control and CUS mice. (D-E) LEfSe analysis; (D) Histogram of the LDA scores (log10) computed for order-level taxa with differential abundance in CTL and CUS mice; (E) Histogram of the LDA scores (log10) computed for genus-level taxa with differential abundance in CTL and CUS mice. N = 3,3.

Mice subjected to the CUS protocol exhibited an increase in alpha diversity according to the Chao1 index (p = 0.0387; t = −3.1617; Fig. 5A), with no significant differences observed in beta diversity according to the Bray-Curtis index (p = 0.2; F = 1.12869; Fig. 5B), possibly due to the small sample size and one sample displaying an atypical pattern. The genus-level relative abundance is illustrated in Fig. 5C, and the Linear Discriminant Analysis Effect Size (LEfSe; considering a p-value cut-off of 0.1 and Log LDA of 2.0)Ganju et al. (2016) indicated that the orders *Lactobacillales* and *Erysipelotrichales* increased in control animals, while the order *Peptostreptococcales_Tissierellales* increased in CUS animals (Fig. 5D). At the genus level, LEfSe showed that the CUS group exhibits an increase in *Muribaculum*, *Parabacteroides* and *Lachnospiraceae_NK4A136_group*, as well as some unassigned genera in the CUS group (Fig. 5E). Rarefaction curve analysis confirmed that the sequencing depth was sufficient to capture the microbial diversity present, as indicated by curves reaching a plateau, demonstrating adequate sampling depth (Saridi and Abu Mansor, 2023) (Supplementary Figure 1).

These findings indicate that the established CUS protocol was effective in inducing stress-related behaviors in mice, accompanied by neuroplastic abnormalities evidenced by the altered expression of key markers such as Syn1, Olig1, and Sox2 in the prefrontal cortex and hippocampus. Additionally, these behavioral and molecular changes were associated with the development of a distinct microbiome profile, characterized by increased alpha diversity and shifts in bacterial taxa, including elevated levels of *Parabacteroides* and *Lachnospiraceae_NK4A136_group* in the CUS group. These results suggest that chronic stress not only impacts behavioral and molecular markers of neuroplasticity but also reshapes the gut microbiome in a manner that may further influence brain function and behavior.

### 3.2 Fecal microbiota transplantation from CUS donors reproduces the stress-associated behavioral, molecular, and microbiota alterations in adult mice

**Fig. 6:**
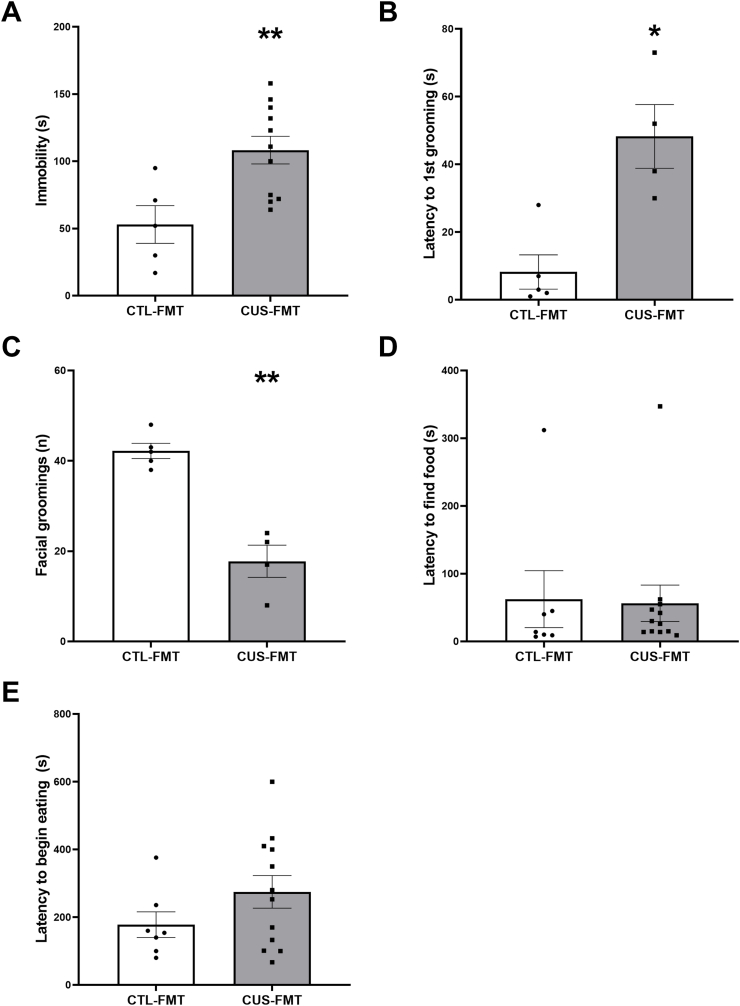
Effect of the CUS protocol on behaviors related to depressive-like behaviors in microbiota receptors mice. (A) Tail Suspension test; A = Immobility (s). (B-C) Sucrose Spray test; B = Latency to first grooming (s); C = number of facial groomings (n). (D-E) Novelty Suppressed Feeding test; D = Latency to find food (s); E = Latency to begin eating (s). Bars represent M±SEM; individual data points are shown. * Indicates P < 0.05, ** indicates P < 0.01. N Tail Suspension; Novelty Suppressed Feeding = 7-12; N Sucrose Spray = 4-5.

Like the donor animals, mice receiving microbiota from CUS donors exhibited increased immobility time in the TS test (p = 0.0082) compared to the control group (Fig. 6A), which received microbiota from non-stressed animals. In the SS test, subjects receiving CUS microbiota showed increased latency to perform the first facial grooming (p = 0.0357) and a decrease in grooming behavior (p = 0.0014) compared to animals that received the control microbiota (Fig. 6B and C). In the NSF test, as observed with the donors, animals in the CUS-FMT group showed a trend to increase the latency to start eating compared to CTL-FMT animals (Fig. 6D and E).

**Fig. 7:**
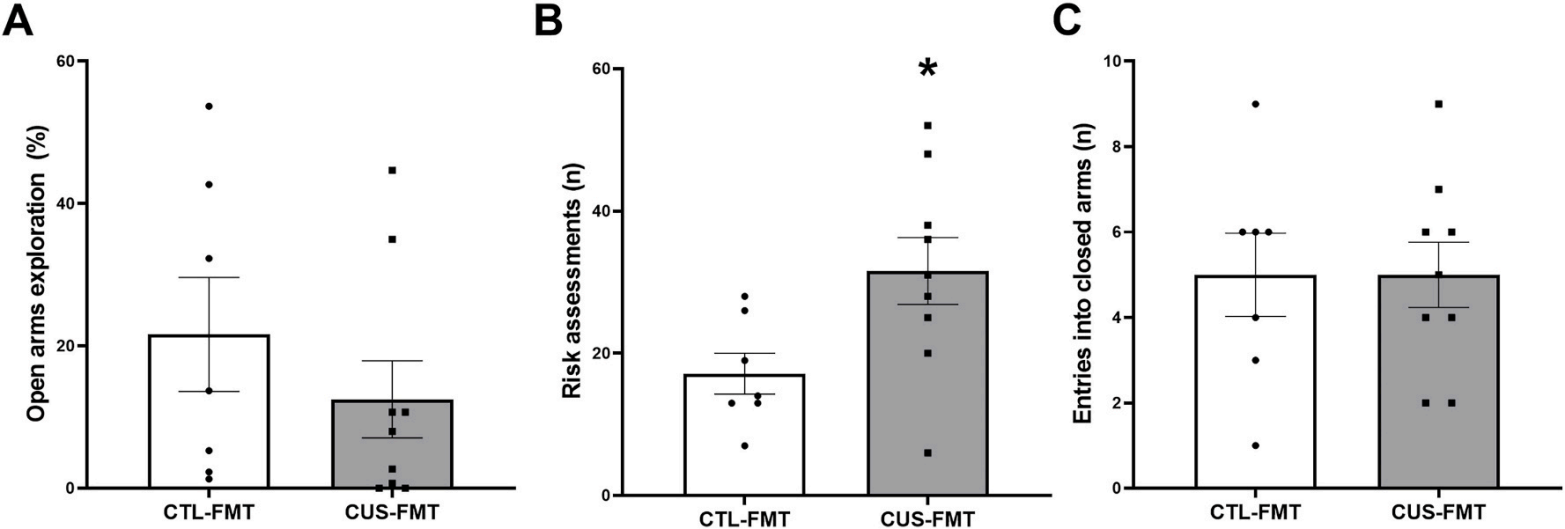
Effect of the CUS protocol on behaviors related to anxiety behaviors in microbiota recipient mice. (A-C) Elevated Plus Maze test; A = Open arms exploration time (%); B = Risk assessment (n); C = Entries into closed arms (n). Bars represent M±SEM; individual data points are shown. * Indicates P < 0.05, **** indicates P < 0.0001. N = 7-12.

In the EPM, a trend for a reduction in open arm exploration time was observed in the CUS-FMT group compared to CTL-FMT (Fig. 7A). Like the donor animals, risk assessment behavior increased (p = 0.029) compared to control-FMT animals (Fig. 7B). There was no difference in the number of closed arm entries (Fig. 7C), suggesting no motor impairment in the animals assessed.

**Fig. 8:**
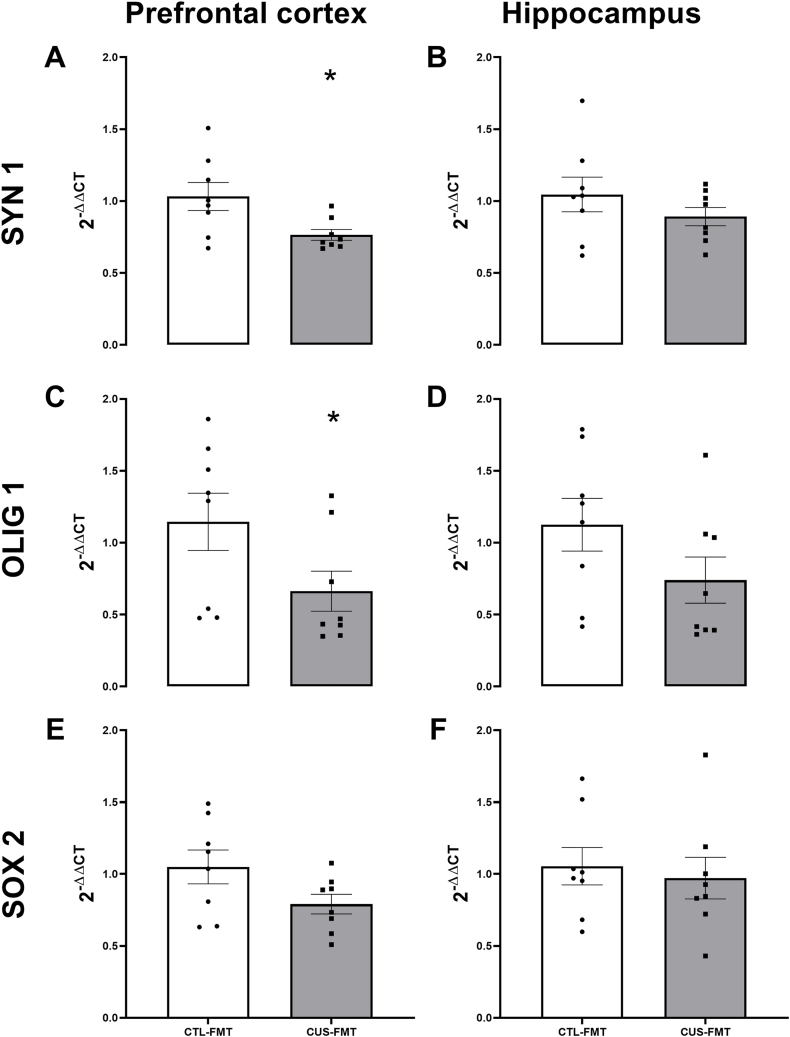
Effect of the FMT protocol on cortical and hippocampal gene expression in microbiota receptors mice. (A-B) Synapsin 1 (Syn1) expression (2-ΔΔCT). (C-D) Oligodendrocyte Transcription Factor 1 (Olig1) expression (2-ΔΔCT). (E-F) SRY-box 2 (Sox2) expression (2-ΔΔCT). Bars represent M±SEM; individual data points are shown. * indicates P < 0.05. N = 8,8.

Animals from the CUS-FMT group exhibited a reduced expression of Syn1 (p = 0.0228) and Olig1 (p = 0.0207) mRNA in the prefrontal cortex compared to those from the control-FMT group, consistent with findings in the respective donor groups. There were no significant changes in the expression of the analyzed genes in the hippocampus (Fig. 8).

**Fig. 9:**
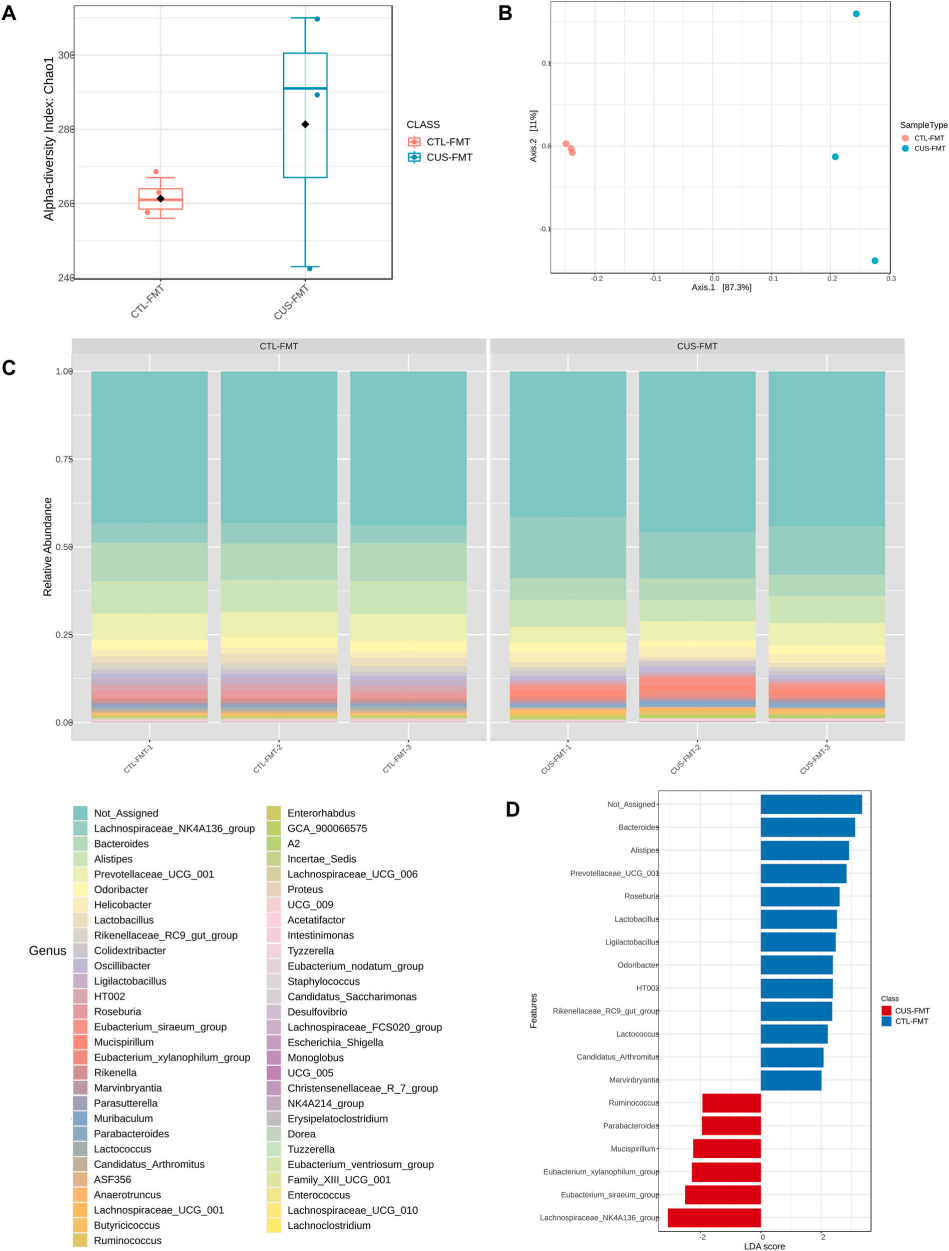
Effect of the FMT protocol on the fecal microbiota composition in recipient mice. (A) Alpha diversity according to the Chao1 index. (B) Beta diversity expressed in the Principal Coordinate Analysis (PCoA) plot with Bray-Curtis dissimilarity. (C) Stacked bar charts showing the actual relative abundance of bacterial genera in Control and CUS mice. (D) LEfSe analysis; Histogram of the LDA scores (log10) computed for genus-level taxa with differential abundance in CTL-FMT and CUS-FMT mice. N = 3,3.

Mice that received microbiota from stressed donors showed no difference in alpha (p = 0.4219; t = −0.99069; Fig. 9A) or beta diversity (p = 0.1; F = 25.223; Fig. 9B) compared to those that received microbiota from healthy donors, possibly due to the small sample size and one sample displaying an atypical pattern. The relative abundance at the genus level is illustrated in Fig. 9C, and the Linear Discriminant Analysis Effect Size showed that the fecal microbiota transplantation protocol altered several genera. The group that received transplants from healthy individuals exhibited an increase in the genera *Bacteroides*, *Alistipes*, *Prevotellaceae_UCG_001*, *Roseburia*, *Lactobacillus*, *Ligilactobacillus*, *Odoribacter*, *HT002*, *Rikenellaceae_RC9_gut_group*, *Lactococcus*, *Candidatus_Arthromitus*, and *Marvinbryantia*. Meanwhile, the group that received transplants from stressed individuals showed an increase in the genera *Lachnospiraceae_NK4A136_group*, *Eubacterium_siraeum_group*, *Eubacterium_xylanophilum_group*, *Mucispirillum*, *Parabacteroides*, and *Ruminococcus*. According to the obtained rarefaction curve, the sequencing depth was sufficient to capture the microbial diversity of the sample (Supplementary Figure 2).

**Fig. 10:**
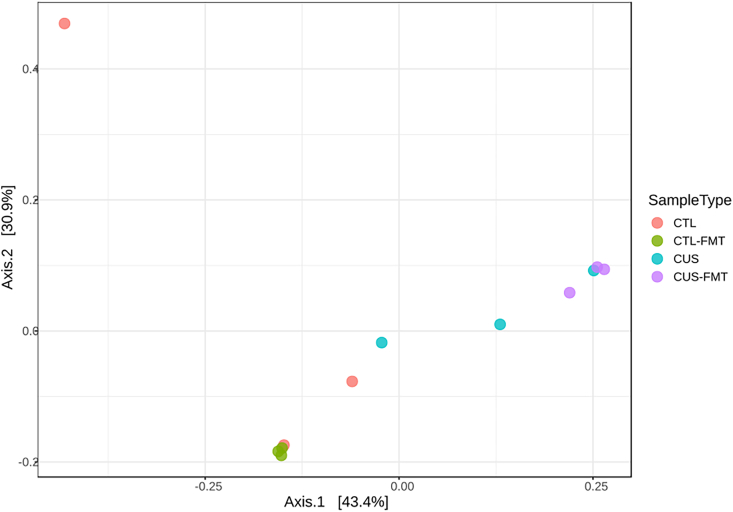
Beta diversity expressed in the Principal Coordinate Analysis (PCoA) plot with Bray-Curtis dissimilarity among the four groups. N = 3 per group. CTL: Control group; CUS: Chronic Unpredictable Stress group; CTL-FMT: Animals that received fecal microbiota transplantation from the control group; CUS-FMT: Animals that received fecal microbiota transplantation from the CUS group.

Beta diversity analysis using the Bray-Curtis dissimilarity index revealed differences in fecal microbiota composition among the CUS, CTL, CUS-FMT, and CTL-FMT groups (F-value: 2.4094; R-squared: 0.47466; p-value: 0.007, Fig. 10). PERMANOVA analysis did not identify specific group differences, potentially due to the small sample size and the presence of one outlier sample. Notably, donor and respective recipient animals clustered together, with minor differences (corroborated by LEfSe analyses), possibly attributable to sample processing and the gavage procedure, as previously discussed (Wilson et al., 2019; Kazemian et al., 2020). This clustering suggests that the FMT protocol successfully induced donor-like profiles in the recipients.

These results demonstrate that FMT from CUS-exposed donors successfully reproduced stress-related behavioral impairments in recipient mice, mirroring depressive-like and anxiety-like behaviors observed in donor animals. Additionally, similar molecular alterations were detected in the recipients, with reduced expression of Syn1 and Olig1 in the prefrontal cortex, aligning with the molecular impairments observed in the original CUS group. Lastly, recipients of CUS microbiota exhibited notable shifts in their gut microbiome profile, with an increase in stress-related genera such as *Lachnospiraceae_NK4A136_group* and *Parabacteroides*, indicating that the FMT protocol effectively induced a shift in microbiota composition consistent with stress-related microbial changes.

## 4 Discussion

The recent development of sequencing methods and metagenomic characterization of microorganisms in biological samples has enabled comprehensive investigations into the complexities of the gut-brain axis and the influence of enteric microbiota on different CNS conditions (Wu et al., 2024). In this context, our study delved into the role of distinct classes of gut-resident microorganisms in the emergence and progression of stress-related symptoms.

Initially, it was confirmed that the CUS protocol could induce stress-related behavioral changes, as previously outlined by Monteiro et al. (2015). The behavioral effects of CUS were reflected in the animal's microbiome. CUS altered the animals' microbiota profile, with increases in the genus *Parabacteroides* and the family Lachnospiraceae. The genus *Parabacteroides*, known for its GABA-producing species, has previously been linked to behavioral impairments associated with mood disorders (Gomez-Nguyen et al., 2021). For example, in 2021, Gomez-Nguyen showed that administering *Parabacteroides distasonis* to behaviorally unimpaired SAMP1 mice induced stress-related behavior in the tail suspension test (Gomez-Nguyen et al., 2021). Similar increases have also been observed in patients diagnosed with MDD (Jiang et al., 2015), and the family *Lachnospiraceae* has been significantly correlated with distinct inflammatory factors in patients with MDD (Bai et al., 2022).

Two orders, *Erysipelotrichales* and *Lactobacillales*, were reduced in stressed animals compared to controls. The *Lactobacillales* group has garnered special attention for its known capacity to modulate mouse behavior through vagal mechanisms (Sgritta et al., 2019), serotonergic neurotransmission (Liu et al., 2016), and the immune system (Ma et al., 2024). Members of this order, such as the species *L. reuteri*, can reverse social impairments in mice in a vagal-dependent manner, involving the oxytocin-dopamine system in the ventral tegmental area (Sgritta et al., 2019). Moreover, treatments with both *L. helveticus* and *L. reuteri* have been capable of increasing serotonin levels in the gut, brain, and hippocampus in animal models, improving stress-related behavior (Ma et al., 2024; Liang et al., 2015). Lastly, in 2021, Qiu and colleagues demonstrated that the species *L. delbrueckii* could mitigate stress-related behavior in mice treated with LPS by inhibiting Toll-Like-4 receptors, which mediated excessive microglial activation (Qiu et al., 2021). Whether the reduction of *Lactobacillales* observed in our CUS animals and FMT recipients contributes to the behavioral phenotypes via any of these mechanisms remains to be determined, as the present study did not directly investigate gut-brain signaling pathways.

The investigation of stress effects on CNS oligodendrogenesis, neurogenesis, and neuroplasticity markers revealed reduced cortical and hippocampal gene expressions of Syn1 and Olig1. Synapsin 1, a predominant neuronal phosphoprotein connected to the actin filaments of the cytoskeleton, acts as an anchoring protein for neurotransmitter-carrying vesicles in the presynaptic neuron (Hilfiker et al., 1999). Since the critical role of Syn1 in the growth of presynaptic processes and neuronal plasticity was first established in the mid-1990s, preclinical studies in animals subjected to moderate chronic stress protocols showed that stress caused impairments in synaptogenesis in the prefrontal cortex (Csabai et al., 2018), corroborating clinical studies that demonstrate reduced expression of Syn1 in patients with MDD (Kang et al., 2012). Furthermore, the Olig1 gene, which operates in concert with its homologue Olig2, plays a vital role in the differentiation and myelination processes of oligodendrocytes (Dai et al., 2015), essential for synaptic regulation and neural circuitry, and crucial for remyelination in mice (Zhou and Anderson, 2002). Olig1 knockout mice displayed neurodevelopmental impairments, including deficits in progenitor cell commitment, oligodendrocyte differentiation, and brain myelination, leading to hypomyelination in adulthood (Dai et al., 2015). Moreover, dysregulation in oligodendrocyte lineage cells, including alterations in Olig1 gene expression, has been noted across various mouse models of stress and human MDD (Suri et al., 2023), suggesting a common signature of oligodendrocyte dysfunction in the frontal cortex.

The next step was to investigate whether transplanting this microbiota to non-stressed animals could replicate the effects of stress in the recipients. Following FMT, recipient mice exhibited changes in the relative composition of fecal microbiota and behavioral deficits like those of the donor animals. The fecal transplantation mimicked the effects of stress on the behavior of these animals, leading to impairments in anhedonia-like, despair-like, and anxiety-like behaviors akin to those observed in the donors. Furthermore, the impairments in gene expression induced by the CUS protocol were evident in the prefrontal cortex but not in the hippocampus of adult recipient animals, including reductions in the expression levels of Syn1 and Olig1. The absence of significant hippocampal changes in FMT recipients, in contrast to the molecular alterations observed in CUS donors, suggests that the microbiota-dependent transfer of stress-related molecular phenotypes may preferentially affect the prefrontal cortex. However, the mechanisms underlying this regional selectivity were not investigated in the present study and warrant further exploration.

Regarding the recipient mice microbiota profile, we observed a similar composition of the donors, with an increase in the genus *Lachnospiraceae_NK4A136_group* and a reduction in members of the order *Lactobacillales* in animals that received feces from stressed donors, validating our transplantation model. Additionally, altered groups were observed that were not seen in the donors and may have been selected during the FMT protocol (Chu et al., 2017). Firstly, in recipients of stressed donors (CUS-FMT), we observed an enrichment of the genus *Ruminococcus*. Both preclinical studies, based on different stress models (Wu et al., 2020; Yang et al., 2021), and clinical studies in individuals with MDD (Zhao et al., 2022; Radjabzadeh et al., 2022) have yielded contradictory results regarding this genus, with some reporting increased and others decreased abundance in stressed animals or depressed individuals compared to controls. These discrepancies likely reflect methodological differences in sequencing approaches and taxonomic resolution, heterogeneity across study populations, and the taxonomic complexity of the genus *Ruminococcus* itself, which encompasses multiple species with potentially distinct functional roles. Importantly, 16S rRNA sequencing at genus level, as employed in the present study, does not capture species-level variation, which may explain apparent contradictions across studies.

Two other genera were significantly enriched in animals that received FMT from stressed donors, namely *Mucispirillum* and *Eubacterium*, both having been previously associated with depressive behavior in either animal models (Zhang et al., 2021; Sun et al., 2023) or clinical studies (Radjabzadeh et al., 2022). Additionally, FMT from stressed animals caused a reduction in the genera *Bacteroides*, *Alistipes*, *Lactobacillus*, *Roseburia*, and *Prevotellaceae* in the recipients, which have been previously observed in preclinical and clinical models of MDD (Jiang et al., 2015; Maes et al., 2020; Sanada et al., 2020; Chevalier et al., 2020). Collectively, these data suggest that the enteric microbiota of the adult individual can induce stress-related behavioral impairments through gene expression alterations related to synaptic plasticity and oligodendrogenesis.

Together, these results demonstrate that the CUS protocol effectively induces stress-related behaviors, reductions in the gene expression of Syn1 and Olig1 in the prefrontal cortex, and changes in the intestinal microbiota composition of the animals. Notably, the transplantation of this altered microbiota in healthy animals replicated the behavioral and gene expression phenotypes observed in the stressed donors. These findings are consistent with a microbiota-dependent transfer of stress-associated phenotypes, though the signaling pathway through which the gut microbiota influences the nervous system and behavior remains to be determined, and underscore the critical role of the gut-brain axis in stress response, supporting the potential of microbiota-targeted interventions as therapeutic strategies in the future.

## 5 Limitations

This study has several limitations. First, only male Swiss mice were used, which limits the generalizability of our findings, given the well-established sexual dimorphism in stress responses (Goel et al., 2014), gut microbiota composition (Kim et al., 2020), and gut–brain axis signaling (Jaggar et al., 2020). Second, the absence of an antibiotic-only control group precludes a formal assessment of the antibiotic regimen's independent effects; however, since the antibiotic pre-treatment was identical across CTL-FMT and CUS-FMT groups, it cannot account for the differential phenotypes observed. Third, microbiome analyses relied on pooled fecal samples collected at the cage level (n = 3 cages per group), which reduces statistical power and precludes individual-level microbiota–behavior correlation analyses, even though behavioral and molecular readouts were performed at the individual animal level (n = 7–21). Finally, fecal suspensions were prepared under aerobic conditions, which may have selected against oxygen-sensitive obligate anaerobes, although successful transfer of strictly anaerobic taxa (e.g., *Lachnospiraceae, Parabacteroides*) was confirmed by 16S rRNA analysis. Future studies should incorporate both sexes, individual-level fecal sampling, larger biological replicates, dedicated antibiotic-only control groups, and strictly anaerobic processing.

The present study was designed as an exploratory investigation, aimed at testing whether FMT performed under simplified experimental conditions could mimic the behavioral effects of stress and at extending the molecular characterization to neuroplasticity-related genes across distinct brain regions. As such, our findings are descriptive and do not address the underlying gut–brain signaling mechanisms. In particular, the regional selectivity observed (prefrontal but not hippocampal molecular alterations in FMT recipients) remains unexplained at the mechanistic level. Future studies involving fecal and plasma metabolomics (e.g., short-chain fatty acids, tryptophan derivatives, bile acids), immune profiling, and selective vagal manipulation (e.g., subdiaphragmatic vagotomy) will be essential to clarify the signaling routes through which the gut microbiota selectively influences cortical neuroplasticity-related gene expression.

## 6 Conclusion

This study demonstrates that stress-associated behavioral and molecular alterations are transferable via the gut microbiota, with effects observed on markers of synaptic plasticity and oligodendrogenesis in the prefrontal cortex. The mechanisms through which the microbiota exerts these effects remain unresolved and constitute an important direction for future investigation.

## Statement of Ethics

All experimental procedures involving animals were approved by the Animal Research Ethics Committee (CEUA - Fiocruz) of the Oswaldo Cruz Foundation (protocol number L-001/17).

## Consent for publication

Not applicable.

## Availability of data and materials

The datasets generated and analyzed during the current study are available from the corresponding author upon reasonable request.

## Authors' contributions

LH performed the experiments, conducted statistical analyses, and drafted the manuscript. FSG contributed to manuscript writing and critically revised the text. RAM and WAS conducted the 16S rRNA sequencing analysis. FRF designed the experimental setup and contributed to the writing and interpretation of the results. All authors read and approved the final manuscript.

## Funding

This work was supported by the São Paulo Research Foundation (FAPESP; grants 2023/16731-6, 2017/03685-3, and 2023/16182-2) and the National Council for Scientific and Technological Development (CNPq; grant 403318/2023-8). We are grateful to these funding agencies for their financial support, which made this study possible.

## CRediT authorship contribution statement

**Lucas Hassib:** Data curation, Formal analysis, Investigation, Methodology, Writing – original draft, Writing – review & editing. **Raphael de Andrade Morraye:** Data curation, Formal analysis. **Wilson Araújo Silva:** Data curation, Formal analysis, Funding acquisition, Supervision, Writing – review & editing. **Francisco Silveira Guimarães:** Data curation, Formal analysis, Writing – review & editing. **Frederico Rogério Ferreira:** Conceptualization, Data curation, Formal analysis, Funding acquisition, Investigation, Methodology, Project administration, Resources, Supervision, Validation, Writing – original draft, Writing – review & editing.

## Declaration of competing interest

The authors declare the following financial interests/personal relationships which may be considered as potential competing interests: Lucas Hassib reports financial support was provided by State of Sao Paulo Research Foundation. Francisco Silveira Guimaraes reports financial support was provided by State of Sao Paulo Research Foundation. Wilson Araujo Silva Jr reports financial support was provided by State of Sao Paulo Research Foundation. Frederico Rogerio Ferreira reports financial support was provided by National Council for Scientific and Technological Development. If there are other authors, they declare that they have no known competing financial interests or personal relationships that could have appeared to influence the work reported in this paper.

## Figures and Tables

**Fig. 1:**
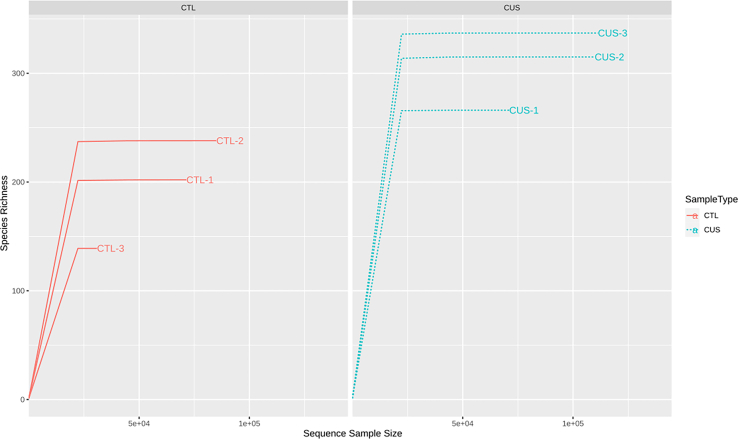
Rarefaction curves for CUS and CTL samples, illustrating the observed diversity relative to the predicted total diversity. The x-axis represents the number of sequences sampled, and the y-axis indicates the species richness detected, as estimated by the Chao1 index.

**Fig. 2:**
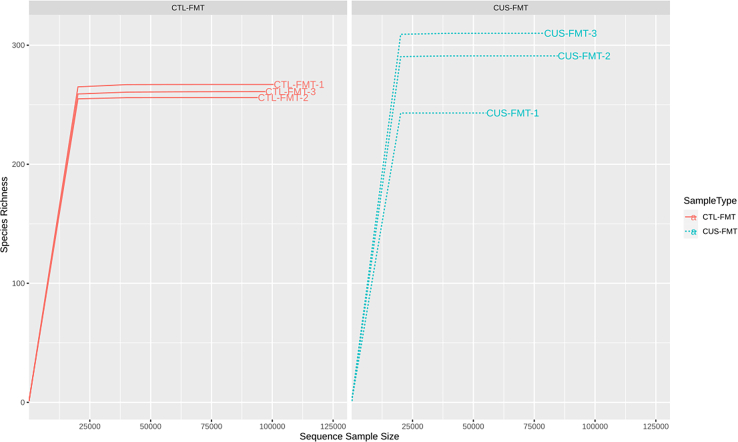
Rarefaction curves for CUS-FMT and CTL-FMT samples, illustrating the observed diversity relative to the predicted total diversity. The x-axis represents the number of sequences sampled, and the y-axis indicates the species richness detected, as estimated by the Chao1 index.

## Data Availability

Data will be made available on request.
